# Qualitative research and nursing knowledge on human responses: considering diagnosis validity

**DOI:** 10.1177/17449871261419692

**Published:** 2026-03-31

**Authors:** Cristina Barradas, Susana Miguel, Sílvia Caldeira

**Affiliations:** PhDc, MSc, RN, Faculty of Health Sciences and Nursing, Universidade Católica Portuguesa, Portugal; PhD, MSc, RN, Centre for Interdisciplinary Research in Health, Faculty of Health Sciences and Nursing, Universidade Católica Portuguesa, Portugal; PhD, MSc, PGDip, RN, Centre for Interdisciplinary Research in Health, Faculty of Health Sciences and Nursing, Universidade Católica Portuguesa, Portugal

## Introduction

In the dynamic and profoundly human realm of nursing, understanding the lived experiences of individuals is essential for delivering compassionate and high-quality care.

Qualitative research plays a crucial role in nursing, providing in-depth insights into patients’ experiences, attitudes and responses to health and illness. This type of research is crucial for comprehending complex human phenomena and informing the development of evidence-based nursing practices (Watson and Jackson, 2025). However, ensuring the validity of qualitative research, particularly in the context of nursing diagnoses, presents several challenges and considerations.

Qualitative research is generally used to explore, describe and understand human experiences (Polit and Beck, 2020). This research paradigm facilitates a way of seeing, understanding, hearing, listening, accessing, and empathetically knowing the other; searching for, examining and interpreting phenomena as they occur in the natural contexts from the participants’ self-perspective (Bayuo et al., 2024). From this point of view, isn’t the nature of qualitative research meeting the deep knowledge towards human responses during life and health processes?

As so, this paper describes a discussion on the contribution of qualitative research to the development of nursing diagnoses. It emphasises the importance of recognising and interpreting human responses to everyday health challenges, responses that are central to nursing practice and critical to accurate, empathetic, and person-centred care plans and integral nursing care.

The discussion is organised into three main sections. The opening section, *Historical Overview*, examines the historical moments that have influenced the development of qualitative research. By acknowledging the genealogy of qualitative methodologies, current practices can be situated within their complex historical trajectories.

The second section, *Qualitative research and the understanding of human responses*, discusses key methodological approaches and highlights the importance of qualitative research in exploring and interpreting human responses to health and illness. These responses are at the core of nursing care and form the basis for nursing diagnoses. Qualitative approaches enable a comprehensive understanding of how individuals, families, and communities experience and respond to health conditions, thereby supporting nurses in delivering individualised, holistic care.

The final section, *Level of Evidence*, addresses the role of qualitative research in enhancing the accuracy and clinical relevance of nursing diagnoses.

## Historical overview

Since the 1940s and 1950s, qualitative research has been widely known through ethnographic studies and action research. However, it formally emerged in the 1960s as a critique of the positivist tradition that dominated research in most disciplines (Denzi and Lincoln, 2018).

From a philosophical perspective, the study of human beings is deeply rooted in the descriptive mode of science. For a long time, Descartes’ vision shaped the research with an idea grounded in objective reality, where the cause-and-effect relationship could explain all events. Nevertheless, Kant is well known for arguing that not all reality is explainable by observation of this relation and that it is essential to explore reality as it is perceived and not just as an observed phenomenon (Hamilton, 1994).Qualitative research has evolved through a series of historical stages described by Denzi and Lincoln (1994). The first one, known as the traditional period (1900–1950), was grounded in the positivist paradigm, with researchers striving for valid, reliable, and objective interpretations of cultural traditions. Subsequently, the modernist or golden age (1950–1970) sought to formalise qualitative methods, aligning them with the rigour of quantitative approaches (Denzi and Lincoln, 1994). The blurred genres phase (1970–1986) introduced intellectual pluralism, as diverse theoretical frameworks coexisted and expanded the scope of qualitative inquiry (Denzi and Lincoln, 1994). This was followed by the crisis of representation (1986–1990), which emphasised reflexivity, subjectivity, and the politics of representation, urging researchers to confront their positionality in the research process (Denzi and Lincoln, 1994). In the postmodern moment (1990–1999), qualitative researchers continued to address the crises of representation and sought new approaches to ethnographic writing. The focus shifted from grand theories and narratives to situation-specific and localised theories (Denzi and Lincoln, 1994).

In 2008, Denzin added three more historical moments. The sixth stage, postexperimental inquiry (1995–2000), characterised by creative and innovative forms of qualitative writing that sought to capture lived experience. The dissemination of such work reinforced its impact. The seventh stage, the methodologically contested present (2000–2004), reflected tensions between traditional objectivist demands for evidence-based methods and interpretive, reflexive approaches (Denzin, 2008). Although critics questioned its scientific rigour, proponents highlighted qualitative research’s unique contribution to understanding complex social phenomena. Finally, the eighth moment (2005–present) situates qualitative inquiry in the contemporary global context, where scholars confront methodological backlash while engaging in critical debates about diversity, freedom, and control in human life (Denzin, 2008).

The concept of qualitative research has taken on different meanings across distinct historical periods, and its contemporary practice must remain attentive to these complex trajectories. Although these phases constitute the legacy of qualitative inquiry, it may be argued that we are in a new stage, marked by intensified philosophical debate, methodological pluralism, and the profound influence of digital and global contexts.

As a result of the historical evolution of qualitative research, it is possible to delineate philosophical assumptions that characterise qualitative research, which are based on ontological, epistemological, axiological and methodological understandings. When considering ontology (the nature of reality), qualitative researchers embrace multiple realities and truths, including those of the researchers and participants, who are the central stance of being and researching human experience (Ravitch, 2021). In epistemological terms (what constitutes valid knowledge and how it is justified), knowledge emerges from participants’ subjective experiences, with the underlying assumption that knowledge is shared (Ravitch, 2021). In axiology (the role of values), qualitative researchers identify, acknowledge and consider their own values and biases, as well as those of the participants, as influential factors in the research process (Ravitch, 2021). Regarding the methodology (the process of conducting the research), it is believed that the research is interpretative; therefore, the reasoning is inductive, with its conclusions progressing from the specific to the general (Ravitch, 2021).

In addition, interpretivism, which emerged in the seventh moment of the historical stages and emphasises the specificity of context and the use of flexible methods and analytical techniques that provide contextualisation, posits that human beings (including both the researcher and the participants) are the primary instruments of a study, and that there is no universal or static truth, but rather multiple perspectives on reality. Context is essential to understanding the individual, group, experience or phenomenon (Ravitch, 2021).

Positionality, which refers to the role of the researcher and their social identity within the research context, is a central issue (Holloway and Wheeler, 2010; Ravitch, 2021). The crisis of representation gave rise to this philosophical assumption, particularly articulated within feminist criticism, which dismantled positivist claims of objectivity and universality. Feminist scholarship underscored the inevitability of researchers’ social identities, values, and power relations shaping the production of knowledge (Erickson, 2018).

## Qualitative research and the understanding of human responses

Several methodological approaches are employed in qualitative research, including action research, ethnography, grounded theory, narrative research and phenomenology (Ravitch, 2021). These different methodologies share common foundations but are distinct in their specific goal and rationale for the analysis. By having different philosophical and epistemological approaches, these methodologies offer the opportunity to find answers to questions centred on the human experience, how it is created and how it gives meaning to life. Understanding how these experiences shape an individual’s reality is a criterion for the development of science and, therefore, is applied in several areas of knowledge (Streubert and Carpenter, 2011).

Interestingly, it can be affirmed that the principles of the qualitative research paradigm align closely with those of the nursing discipline, as both embrace a holistic, person-centred perspective. This approach not only fosters a deeper understanding of human experiences but also enriches nursing practice and science by integrating patient narratives and contextual insights (Al Maqbali, 2024b; Polit and Beck, 2020).

Nurses with a focus on protecting, promoting, and optimising health and human functioning seek to prevent illness and injury, facilitate the healing process and relieve suffering. They aim to understand the broad spectrum of human experiences (American Nurses Association, 2021). In practice, nursing research often arises from questions prompted by these challenges from clinical practice (Al Maqbali, 2024a). In this perspective, nurses are not limited to providing care; they also embrace the role of active researchers, mobilising both knowledge and experience. Through this dual engagement, they aim to recognise human responses arising from health experiences and, more specifically, from actual or potential health problems, to provide individualised and holistic healthcare (Herdman et al., 2024). Human responses reflect how people process and manage their daily health issues, which are complex and influenced by internal factors, as well as cultural, social, and historical contexts (Jiménez Arroyo and Rangel Flores, 2017).

Daily, nurses deal with these responses to health conditions/life processes among individuals, families, groups, and communities, which are the central concern of their care and the central element of their diagnoses (Herdman et al., 2024).

Nursing diagnoses, as a stage of the nursing process, reflect a clinical judgement about a human response to health conditions/life processes or a susceptibility to such a response. It goes beyond observations made by nurses, it is a result of nursing disciplinary knowledge and clinical reasoning applied to assessment data (Herdman et al., 2024). They represent a result of clinical reasoning and decision-making by nurses, which is why diagnostic accuracy is essential, as it supports this decision-making process (Da Silva et al., 2020).

Defined as the judgement regarding the degree of relevance, specificity, and consistency of the existing clues for a given diagnosis, the accuracy of nursing diagnoses shapes the interventions and impacts the effectiveness of the results, through the planning and implementation of nursing interventions with a view to the expected result (Da Silva et al., 2020).

The first stage of the nursing process, the initial assessment, is extremely important in the accuracy of diagnoses because it allows nurses to identify and relate the defining characteristics to related factors and/or risk factors (Kamitsuru and Herdman, 2024). This phase of the nursing process is not a neutral or purely technical act; it involves interpreting patient cues, contexts and meanings, reflecting the principle that knowledge is constructed through interaction and interpretation, much like in qualitative research. At the same time, nurses bring their own professional expertise, cultural background, and personal values to the assessment, underscoring the idea that the identity and role of the nurse or researcher shape the production of knowledge. In both domains, reflexivity is essential for rigour: nurses must remain aware of how their perspectives influence diagnostic accuracy, just as researchers must acknowledge how their positionality shapes data interpretation. Thus, both practices rely on recognising complexity, context, and subjectivity rather than presuming universal objectivity.

## Level of evidence

NANDA-I – a professional organisation that develops and maintains the standardised classification of nursing diagnoses – supports the development of nursing diagnosis criteria to increase the specificity and accuracy of the diagnostic process (Lopes et al., 2024).

However, the long list of diagnostic indicators can be confusing, so in the current developmental stage of nursing diagnoses, identifying nursing diagnostic criteria based on research is an urgent task. Without diagnostic criteria, it is not easy to make an accurate nursing diagnosis (Lopes and Herdman, 2024).

The research results allow the formulation of accurate nursing diagnoses. For this reason, NANDA-I organises nursing diagnoses into levels of evidence. The hierarchy of evidence for the validity of NANDA-I diagnoses is guided by criteria relating to the types of studies that generated them (Lopes et al., 2024). The qualitative validity, level of evidence 2.3.1 in NANDA-I, ‘refers to the degree to which diagnostic interpretation is supported by clinical elements captured from individual subjective experiences’ (Lopes et al., 2024: 142). This approach acknowledges and values human experience, in contrast to traditional evidence hierarchies in which qualitative studies are typically ranked lower.

In contrast to quantitative research, qualitative research does not possess a universally recognised ‘gold standard’ for evaluating quality. Explicit frameworks or guidelines for integrating qualitative evidence into clinical recommendations and practice guidelines are limited, and consensus is lacking on how to grade the quality of qualitative studies or assess the strength of recommendations derived from them (Sekhon et al., 2024).

The systematic review conducted by Sekhon et al. (2024) highlighted the existence of multiple checklists designed to assess the methodological quality of qualitative research. However, no universally applicable hierarchy of evidence has been established to categorise qualitative studies. In an earlier attempt to address this gap, Daly et al. (2007) proposed a hierarchy of evidence for qualitative health research, emphasising the capacity of reported findings to inform practice or policy. At the highest level were generalisable studies, followed by conceptual studies, descriptive studies and, at the lowest level, single case studies.

Regardless of the level of evidence, qualitative research offers a unique way to understand the complexity, subjectivity, and social and political contexts of the health-disease experience, thereby reinforcing nursing’s reliance on qualitative methodologies (Thorne, 2022). A brief exploratory search in PubMed using the descriptors ‘qualitative research’ and ‘nursing’ reveals an increase in qualitative research in nursing, encompassing studies such as phenomenological research, grounded theory, action research, and narrative research.

On the other hand, a scarce number of qualitative studies are found in the development/improvement of nursing diagnoses when conducting an exploratory search also in PubMed using the descriptors ‘nursing diagnosis’ and ‘qualitative research’ and ‘NANDA-I’. For example, Pinto et al. (2017) aimed to contribute to the development of the nursing diagnosis ‘impaired comfort’ as outlined in the NANDA-I. This study provided a broad and valuable insight into the experiences of discomfort, contributing to the achievement of the proposed objective and the advancement of nursing knowledge. The findings contributed to the development of focused, individualised, and effective nursing interventions for people experiencing this diagnosis.

However, the development of nursing diagnoses is essentially based on secondary studies, such as the systematic literature review carried out by Silva et al. (2022) to identify the clinical indicators to determine the presence or absence of the nursing diagnosis ‘ineffective health management’ and, more recently, the protocol of an umbrella review carried out by Monforte-Royo et al. (2024) which aims to define and validate the nursing diagnosis ‘desire to die’ in people with advanced disease.

Qualitative research, by systematically examining human responses, offers a rigorous means of elucidating the complex elements underlying nursing diagnoses and thereby strengthens the foundation of nurses’ clinical reasoning.

## Implications for nursing

Nursing is a discipline defined not only by its scientific foundations but also by its profound engagement with human experience. By prioritising the understanding of individuals in their broader contexts, rather than limiting care to disease management, nursing affirms its distinctive role as a profession attuned to the subjective, relational, and transformative dimensions of health.

Qualitative research does not follow a linear process; rather, it unfolds through continuous interaction and cyclical building of insights. This interactive nature mirrors the complexity of human experience and aligns with nursing’s paradigm of holistic care. Within this framework, qualitative research becomes indispensable; by exploring emotions, transitions, narratives, and meanings, qualitative inquiry illuminates essential aspects of care. It provides nurses with deeper insights into how individuals experience illness, healing and everyday health challenges, thereby strengthening clinical reasoning and enriching nursing diagnoses.

For this reason, nurses can develop accurate diagnoses that support their interventions and impact the results, not only for these communities but also directed to the contexts of their clinical practice and the people they care for.

Qualitative research is not merely a methodological choice in nursing, it is a reflection of the profession’s paradigm, by privileging human experience, context, and meaning. It reinforces nursing’s identity and ensures that clinical reasoning remains both scientifically rigorous and profoundly human.

The implications for nursing are significant. Qualitative research reinforces the profession’s distinct identity, validates its epistemological stance, and ensures that evidence-based practice incorporates not only measurable outcomes but also the human meaning behind them.

## Conclusion

The dynamic process of qualitative research helps to understand patterns of health behaviours, describe experiences, design health interventions, and develop theories that support and sustain care delivery.

The strength of this approach lies in the richness of the data and the in-depth descriptions that can be obtained during data collection, so this method is considered more humanistic and person-centred.

Qualitative research explores, narrates and explains phenomena, making sense of complex reality. The results of this research enable us to understand human responses to health/disease experiences, and to develop interventions, models and explanatory theories fundamental for providing individualised and holistic nursing care that responds to the needs of people, families, groups, or communities. Understanding the richness and complexity of the human experience is the focus of qualitative research and the essence of nursing.
